# Hemosuccus Pancreaticus Following Acute Pancreatitis in a 12-years-old Boy Secondary to Pancreatic Pseudoaneurysm Treated With Endovascular Coil Embolization

**DOI:** 10.1097/PG9.0000000000000125

**Published:** 2021-10-25

**Authors:** Christiana Ekezie, Kara G. Gill, Patrick R. Pfau, Andrew R. Johannes, Michael Woods, Jason Pinchot, Katryn N. Furuya, Daniel M. O’Connell, Thomas Ratchford, Luther Sigurdsson, Dorota Walkiewicz, Alyssa Valentyne, Nicole E. St. Clair, Mary L. Ehlenbach, Therese Woodring, Istvan Danko

**Affiliations:** From the * Department of Pediatrics, University of Wisconsin, School of Medicine and Public Health, Madison, WI; † Division of Pediatric Radiology, Department of Radiology, University of Wisconsin, School of Medicine and Public Health, Madison, WI; ‡ Division of Gastroenterology and Hepatology, Department of Medicine, University of Wisconsin, School of Medicine and Public Health, Madison, WI; § Division of Interventional Radiology, Department of Radiology, University of Wisconsin, School of Medicine and Public Health, Madison, WI; ∥ Division of Pediatric Gastroenterology Hepatology and Nutrition, Department of Pediatrics, University of Wisconsin, School of Medicine and Public Health, Madison, WI; ¶ University of Wisconsin, School of Medicine and Public Health, Madison, WI; # Division of Pediatric Hospital Medicine, Department of Pediatrics, University of Wisconsin, School of Medicine and Public Health, Madison, WI

**Keywords:** hemosuccus pancreaticus, gastrointestinal, bleeding, coil embolization, pancreatitis, pseudoaneurysm

## Abstract

Hemosuccus pancreaticus is a very rare cause of upper gastrointestinal bleeding in children. It is defined as bleeding from the pancreatic or peripancreatic vessels into the main pancreatic duct and may be life-threatening. We present the case of a 12-year-old boy with hematemesis and severe anemia that developed following an episode of acute pancreatitis. Upper endoscopy did not reveal a bleeding source. An endoscopic retrograde cholangiopancreatography performed for the evaluation of common bile duct obstruction identified bleeding from the pancreatic duct. Subsequently, the bleeding source, a pseudoaneurysm of the splenic artery, was identified by conventional angiography and occluded with coil embolization. The diagnosis of hemosuccus pancreaticus may be difficult in children due to rare occurrence and the unusual anatomical site; hence, a high index of suspicion is needed in a patient with a history of pancreatitis who presents with intermittent upper gastrointestinal bleeding and normal upper endoscopy.

## INTRODUCTION

Hemosuccus pancreaticus is a rare, potentially life threatening cause of upper gastrointestinal bleeding. It is defined as bleeding from the pancreatic or peri-pancreatic vessels into the main pancreatic duct (1). It occurs mainly in adults, accounting for about 1 of 1500 cases of upper gastrointestinal bleeding (2). Only a few cases have been reported in children, all associated with pancreatitis (3–5). To our knowledge, no cases have been reported in the pediatric literature from the United States.

## CASE REPORT

Patient is a 12-year-old male with a complex medical history including TUBA1A mutation, cerebral palsy with spastic quadriplegia, and gastrojejunal tube feed dependence. He was readmitted for hematemesis and anemia requiring transfusion after a recent hospitalization for acute pancreatitis 12 weeks after spinal fusion surgery. This was his first known episode of acute pancreatitis. The parents of the patient provided informed consent for publication of the case details.

On admission, gastric lavage showed clear fluid, without visible blood. Contrast enhanced CT of the abdomen demonstrated acute pancreatitis with multifocal acute necrotic collections (ANCs) in and around the pancreas, thrombosis of the main portal and proximal superior mesenteric vein, and antral wall thickening. This and subsequent imaging studies did not indicate chronic pancreatitis. Esophagogastroduodenoscopy (EGD) showed gastritis, duodenitis without evidence of bleeding. He was started on pantoprazole, sucralfate, enoxaparin, TPN, and later on broad-spectrum antibiotics for intermittent fevers. For the next 2 weeks epigastric tenderness, abdominal distension, nonbloody emesis persisted with varying severity; amylase and lipase were trending down and liver enzymes remained normal. Repeat CT scans did not reveal abnormalities in the pancreatic duct or contrast within the duodenum to suggest active bleeding.

**FIGURE 1.**
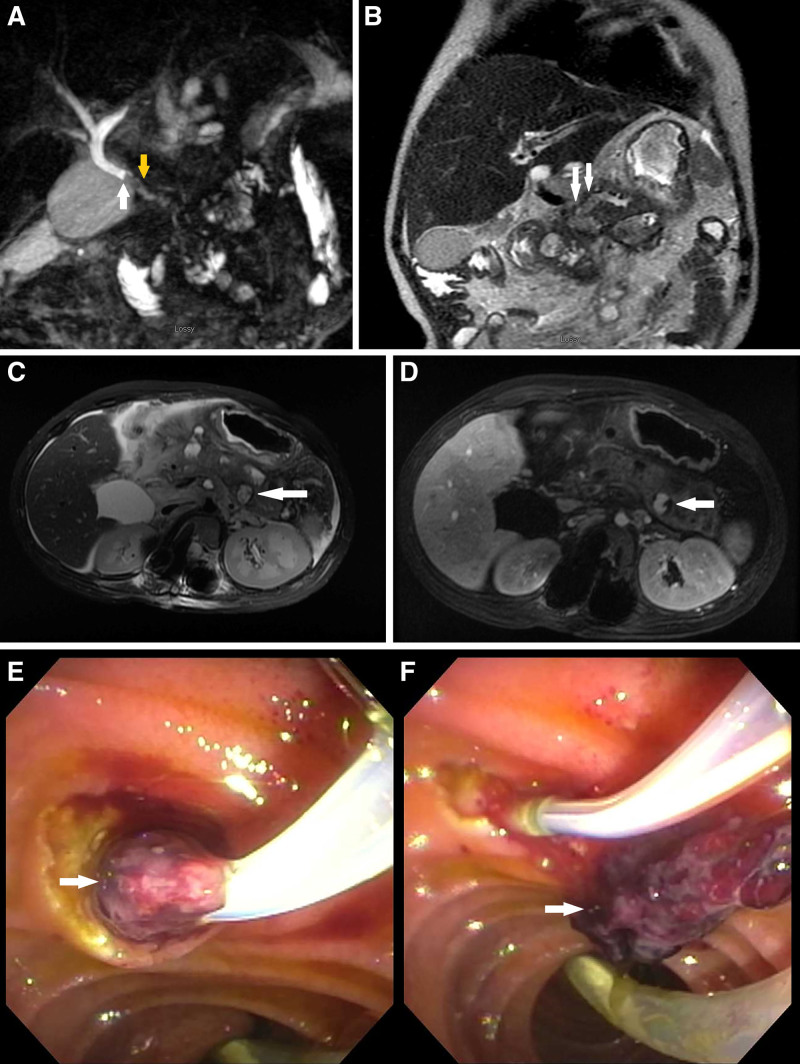
Demonstration of bleeding into the pancreatic duct by MRCP and ERCP. A) 3D coronal, heavily T2-weighted reformatted MIP from MRCP. Arrows point at an area with reduced signal intensity within the distal common bile duct consistent with blood/blood clot. B) Coronal T2-weighted MR image of abdomen. Arrows point at dilated main pancreatic duct. The intraductal fluid is less hyperintense than cerebrospinal fluid indicating that it is more complex/proteinaceous, potentially blood; however, the signal characteristics of the fluid are not specific to hemorrhage. There was no bright intraductal signal on T1 weighted images, indicating that the patient was not actively bleeding into the pancreatic duct at the time of the exam (not shown). C) Axial fat-saturated T2-weighted MR image of the abdomen shows intrapancreatic and peripancreatic fluid collections. The arrow points to the intraparenchymal pseudoaneurysm and demonstrates mild heterogenous signal. D) The fluid collection in the tail of the pancreas fills with contrast on arterial phase imaging consistent with pseudoaneurysm. The arrow points at the area with high signal intensity within the intraparenchymal collection. There was no T1 hyperintensity within the pseudoaneurysm on pre-contrast T1-weighted imaging (not shown). E) and F) ERCP images of blood clot in the papilla Vateri and in the duodenal lumen, respectively (arrows). ERCP = endoscopic retrograde cholangiopancreatography; MIP, maximum intensity projection; MRCP = magnetic resonance cholangiopancreatography.

**FIGURE 2.**
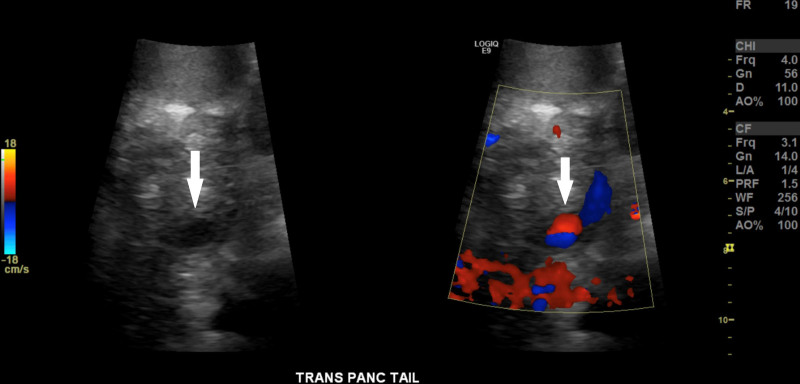
Demonstration of pseudoaneurysm by Doppler ultrasound. Side-by-side gray scale and color Doppler image of the pancreatic tail reveal a hypoechoic area that fills with color in a “yin yang” pattern classic for pseudoaneurysm (arrows).

**FIGURE 3.**
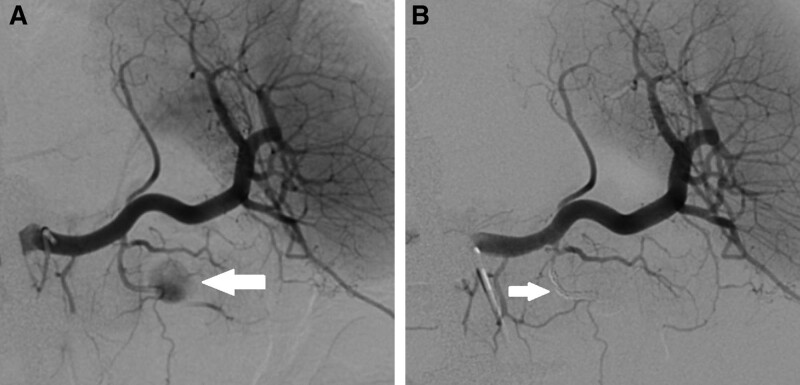
Demonstration of successful coil embolization by angiography. A) Conventional splenic artery angiography confirmed a pseudoaneurysm arising from the transverse pancreatic artery off the pancreatic magna artery (arrow). B) The pseudoaneurysm no longer fills with contrast after coil embolization (arrow).

On hospital day 26, gastric content turned bright red with blood prompting a hold of enoxaparin. He had melena for the next several days without further evidence of gastric bleeding. During this same period, he developed a direct hyperbilirubinemia and a sudden rise in previously normal aminotransferases and alkaline phosphatase. Magnetic resonance cholangiopancreatography and dynamic angiography with and without contrast (MRCP/MRA) showed the ANCs increasing in size, sludge-filled gallbladder, sludge within the common bile duct, dilation of the central intrahepatic, proper hepatic and distal common bile ducts, debris filled dilated distal pancreatic duct compressing the common bile duct, stable nonocclusive thrombus in the proximal portal vein, and continued occlusion of the distal superior mesenteric vein (Fig. 1A, B). Dynamic MRA demonstrated focal contrast accumulation within a 1.6-cm ANC near the junction of the pancreatic body and tail, corresponding to a pancreatic pseudoaneurysm (Fig. 1C, D). Patient underwent EGD and endoscopic retrograde cholangiopancreatography (ERCP) the following day. EGD demonstrated no active bleeding. ERCP demonstrated debris within the biliary tree and blood clot in the ampulla of Vater (Fig. 1E, F), confirming the diagnosis of hemosuccus pancreaticus. A stent was placed into the ventral pancreatic duct. On hospital day 31, the patient had another episode of melena prompting imaging. Abdominal ultrasound confirmed a pancreatic pseudoaneurysm within the pancreatic tail (Fig. 2). Conventional splenic angiography showed a pseudoaneurysm arising from the transverse pancreatic artery off the pancreatic magna artery (Fig. 3A). This was treated with coil embolization of the artery both before and after the pseudoaneurysm (Fig. 3B). The patient improved clinically with no additional episodes of hematemesis, hematochezia, or melena.

## DISCUSSION

In acute pancreatitis, bleeding may occur from gastrointestinal ulcers and varices, or from erosion and rupture of vessels caused by pancreatic inflammation, necrosis, abscess, or pseudocyst, which may occur with or without pseudoaneurysm formation. There are no large-scale studies on the incidence of hemorrhagic complications in pediatric acute pancreatitis. The reported incidence in adults varies widely between 1% and 23% (6). In 2 large adult series 17% and 33% of significant bleeding complications of acute pancreatitis were related to pseudoaneurysms (6, 7). Bleeding from pseudoaneurysms is more common in chronic compared to acute pancreatitis and was reported to occur in 7.1% versus 3.2% of patients respectively in a large recent adult retrospective study (8).

The diagnosis of hemosuccus pancreaticus can be difficult considering the rarity and the anatomical site; hence, a high index of suspicion is needed for a patient with a history of pancreatitis who presents with intermittent gastrointestinal bleeding. An EGD may rule out other causes like peptic ulcers, gastritis, or gastric or esophageal varices and may show bleeding from the papilla. The bleeding may be intermittent due to clot formation in the main duct (5) and may not be observed on EGD, as in our patient. Doppler ultrasound can visualize ANCs and pseudoaneurysms of the arteries; however, it may be limited by restricted sonographic windows and operator dependence (9). Contrast-enhanced CT or MRI may demonstrate pancreatitis, pancreatic necrosis, ANCs, and pseudoaneurysm, but detection may be limited by the lack of an arteriographic phase if not obtained. A noncontrast CT may show intermediate to high attenuation fluid in the pancreatic duct indicating that the bleeding is coming from the pancreas (10). Of all the imaging modalities, conventional angiography is the diagnostic reference standard. It can identify the artery involved as well as pseudoaneurysms and delineates the anatomy for therapeutic intervention (9).

In retrospect, in our patient, the pancreatic duct was mildly dilated on the initial MRI, which could have been an early diagnostic clue. However, artifacts in this region on the 2 subsequent CT scans related to the patient’s spinal hardware made it difficult to visualize the pancreatic duct well and to make a good comparison between the 2 imaging modalities. Furthermore, these CT scans did not otherwise indicate active bleeding, albeit the gastrojejunal tube in place in the patient at the time may have obscured small amounts of high attenuation hemorrhage in the duodenum. The pancreatic duct did become more dilated between the MRI studies performed 14 days apart, providing another potential clue to search for pseudoaneurysm and hemorrhage into the duct, although such dilation could occur for other reasons, including obstruction caused by pancreatic head edema or fluid collection near the pancreatic head.

The most common pathomechanism of hemosuccus pancreaticus is rupture of a pseudoaneurysm, which develops by erosion of vessel walls as a result of pancreatitis (8, 11). Less common causes include tumors, vascular malformations, or iatrogenic causes related to procedures. The splenic artery is involved in about 60% of cases, including in our patient. The gastroduodenal, pancreaticoduodenal, and hepatic arteries may also be involved. Patients may present with melena, hematemesis, and anemia (5). Other symptoms include intermittent epigastric pain, elevation of liver enzymes, jaundice (likely related to obstruction of the common bile duct and pancreatic duct by blood), nausea, and nonbloody emesis.

The treatment in hemodynamically stable patients is occlusion of the pseudoaneurysm with coil embolization, balloon tamponade, and stent grafting by interventional radiology (10). Coil embolization is successful in 79%–100% of cases and often produces immediate results as seen in our patient. Surgical treatment is indicated in uncontrolled hemorrhage, when embolization is not feasible or has failed (9). In most cases, this involves excision of the pseudoaneurysm and pseudocysts (10). Ligation of the arteries may be attempted but the risk of recurrence is high. The most aggressive approach is pancreatic resection (9, 10).

In conclusion, hemosuccus pancreaticus is a very rare cause of upper GI bleeding in children. There should be a high index of suspicion in a patient with a history of pancreatitis who presents with intermittent upper GI bleeding from no other identifiable anatomical site. In such patients, conventional angiography should be considered and may be life-saving.
